# Multiple Myeloma and Change of ABO Blood Group Type: A Case Report

**DOI:** 10.7759/cureus.10654

**Published:** 2020-09-25

**Authors:** Madeeha Subhan Waleed, Waleed Sadiq

**Affiliations:** 1 Internal Medicine, Ayub Teaching Hospital, Islamabad, PAK; 2 Internal Medicine, Staten Island University Hospital, New York, USA

**Keywords:** tumor, hematology, hypercalcemia, multiple myeloma, malignancy, blood group

## Abstract

Multiple myeloma is a hematopoietic stem cell malignancy that involves the plasma cells. It starts insidiously and usually involves males in their 60's. Clinical manifestations usually include anemia, kidney disease, hypercalcemia, and bone pains. We present a male with multiple myeloma whose blood group changed from AB positive to O positive. ABO blood group change can occur in multiple myeloma so blood group should be checked thoroughly in patients with hematological malignancies to prevent serious hematological reactions.

## Introduction

Multiple myeloma is a hematopoietic stem cell malignancy that involves the plasma cells. It starts insidiously and usually involves males in their 60's. Clinical manifestations usually include anemia, kidney disease, hypercalcemia, and bone pains. Neurological manifestations secondary to amyloidosis can occur as well. Diagnosis involves rouleaux formation on a peripheral smear, positive paraproteinemia on urine or serum electrophoresis, skewing of kappa to lambda ratio, and more than 10% plasma cells on a bone-marrow biopsy. Complications include sepsis, hyperviscosity syndrome, renal insufficiency, and side effects of chemotherapy use. We present to you a case of transfusion-dependent anemia secondary to multiple myeloma leading to the conversion of the blood group from AB positive to O positive subtype. Multiple myeloma makes up to 10% of all hematological malignancies [1,2]. Three percent of the population above the age of 50 have monoclonal gammopathy of undetermined significance (MGUS) that can further turn into a malignancy at a rate of approximately 1% per year [3,4]. Blood group antigens adhere to the red blood cell membrane and do not change throughout an individual's lifetime. Change of blood group type in multiple myeloma is a rare occurrence.

## Case presentation

A 59-year-old male with a past medical history of multiple myeloma (MM) presented to the emergency department with shortness of breath, fatigue, and palpitations for about two weeks. These symptoms were progressive. They improved at rest and worsened during walking. The patient denied fever, mucosal bleeding, or blurred vision. A review of systems was unremarkable. He was diagnosed with MM two years ago using a bone-marrow biopsy which showed more than 65% plasma cells. Fluorescence in situ hybridization (FISH) analysis revealed hypodiploidy, i.e. t (11;14) translocation. His current medications were thalidomide, dexamethasone, bortezomib, and alendronate. The patient also received 26 units of the packed red blood cell concentrate in the past eight months due to recurrent severe anemia. Regarding his social history, the patient was married with three children. He was a nonsmoker and nonalcoholic. On examination, the patient was oriented to time, place, and person. Conjunctivae were markedly pale and mucous membranes were dry. The cardiovascular exam was positive for hyperdynamic circulation and positive S3 sound. Lungs were clear bilaterally. Bowel sounds were hypoactive. The tip of the liver was palpated three fingerbreadths below the costal margin and splenomegaly was present. The patient was cross-matched and started on O negative packed red blood cells and conservative fluid resuscitation. Chelation with deferoxamine was initiated later as ferritin levels were greater than 1,000 ng/mL leading to secondary hemochromatosis. After eight days of chelation therapy, his ferritin levels decreased to 210 ng/mL. The rest of the blood results, electrolytes, electrocardiogram, and chest X-Ray were normal ruling out cardiovascular or respiratory causes of dyspnea and palpitations. His complete blood picture, renal, and liver function tests are shown in Table 1 and Table 2.

**Table 1:** Complete blood picture

	Values	Normal Values
Hemoglobin	7.5 g/dl	11-18 g/dl
White blood cells	4000/uL	4500/uL
Mean corpuscular volume	70 fL	80-99 fL
Haematocrit	22%	35-60%
Total iron-binding capacity	200 ug/dl	250-300 ug/dl

**Table 2:** Renal and liver function tests

	Values	Normal values
Alanine transferase	19 U/L	10-50 U/L
Urea	119 mg/dL	10-50 mg/dL
Creatine	1.9 mg/dL	0.4-1.4 mg/dL
Alkaline phosphatase	122 U/L	80-360 U/L
Total bilirubin	0.1 mg/dL	0.3-1.3 mg/dL
Uric acid	8.6 mg/dL	3.0-8.0 mg/dL

His serum protein electrophoresis showed a sharp well-defined peak in the gamma region, consistent with a monoclonal gammopathy. The results of serum protein electrophoresis are shown in Table 3.

**Table 3:** Serum protein electrophoresis

	Current result Units:g/dL	Normal range Units:g/dL
Total protein	14.7	
Albumin	4.9	6.4-8.3
Alpha 1	0.2	3.2-5.5
Alpha 2	0.9	0.4-1.2
Beta	1.0	0.5-1.1
Gamma	7.5	0.5-1.6
Alpha/Gamma Ratio	0.5	1.2-1.7
Kappa light chain	135	6.2-13.5
Lambda light chain	0.8	3.1-7.2

## Discussion

Multiple myeloma accounts for 10-20% of all hematological malignancies [5]. It has an annual incidence of 6/100000 in Western countries [6]. The most common hematological malignancy is Hodgkin lymphoma and the second is multiple myeloma. Multiple myeloma occurs more frequently in males. The median age of patients at diagnosis is 66 years [7]. In various malignancies, especially hematological malignancies, the red blood cell antigen might undergo variation. The most commonly involved antigen undergoing variation is the ABO antigen [8-11]. Acute myelogenous leukemia accounts for the most common cause of ABO antigen variation. Our case had blood group AB positive since birth; after being diagnosed with multiple myeloma and undergoing multiple blood transfusions, his blood group changed to O positive. A, B, and H antigens are formed from the same precursor substance. The production of ABO antigens depends on the functioning of two glycosyltransferases. The first enzyme, H transferase, adds L-fucose to the terminal galactose of the precursor substance. The H substance is then acted on by the A or B transferases that add an N-acetyl galactosamine or galactose, respectively. There are two possible mechanisms for the weakening of ABO antigens in hematopoietic diseases. The first mechanism is the inactivation of A/B transferases, and the second is the inactivation of H transferase [9-15].

## Conclusions

ABO blood group change can occur in multiple myelomas, so the blood group should be checked thoroughly in patients with hematological malignancies. Mismatched blood transfusion can be fatal.
